# Tailoring Mathematical Models to Stem-Cell Derived Cardiomyocyte Lines Can Improve Predictions of Drug-Induced Changes to Their Electrophysiology

**DOI:** 10.3389/fphys.2017.00986

**Published:** 2017-12-12

**Authors:** Chon Lok Lei, Ken Wang, Michael Clerx, Ross H. Johnstone, Maria P. Hortigon-Vinagre, Victor Zamora, Andrew Allan, Godfrey L. Smith, David J. Gavaghan, Gary R. Mirams, Liudmila Polonchuk

**Affiliations:** ^1^Computational Biology, Department of Computer Science, University of Oxford, Oxford, United Kingdom; ^2^Roche Pharma Research and Early Development, Roche Innovation Center Basel, F. Hoffmann-La Roche Ltd., Basel, Switzerland; ^3^Clyde Biosciences, BioCity Scotland, Newhouse, United Kingdom; ^4^Centre for Mathematical Medicine and Biology, School of Mathematical Sciences, University of Nottingham, Nottingham, United Kingdom

**Keywords:** cardiomyocytes, stem cell derived, electrophysiology, mathematical model, pharmacology, variability, computational model

## Abstract

Human induced pluripotent stem cell derived cardiomyocytes (iPSC-CMs) have applications in disease modeling, cell therapy, drug screening and personalized medicine. Computational models can be used to interpret experimental findings in iPSC-CMs, provide mechanistic insights, and translate these findings to adult cardiomyocyte (CM) electrophysiology. However, different cell lines display different expression of ion channels, pumps and receptors, and show differences in electrophysiology. In this exploratory study, we use a mathematical model based on iPSC-CMs from Cellular Dynamic International (CDI, iCell), and compare its predictions to novel experimental recordings made with the Axiogenesis Cor.4U line. We show that tailoring this model to the specific cell line, even using limited data and a relatively simple approach, leads to improved predictions of baseline behavior and response to drugs. This demonstrates the need and the feasibility to tailor models to individual cell lines, although a more refined approach will be needed to characterize individual currents, address differences in ion current kinetics, and further improve these results.

## 1. Introduction

Induced pluripotent stem cells (iPSCs) can be generated by harvesting fully differentiated and mature somatic cells from donors and reprogramming them to the pluripotent state (Takahashi et al., 2007; Yu et al., 2007). From this state, similarly to embryonic stem cells (ESCs), iPSCs can be differentiated into cell types used for drug screening, disease modeling, cell therapy, and testing of personalized treatments (Robinton and Daley, 2012; Shi et al., 2017). But unlike ESCs, iPSCs are harvested from mature donors, which greatly increases their availability, can provide patient-specific cells, and avoids ethical issues associated with the use of embryonic cells (Holm, 2008). Compared to animal *ex-vivo* cell models, iPSCs avoid issues of inter-species differences in protein expression and cellular physiology (Houser et al., 2012; Milani-Nejad and Janssen, 2014).

Differentiation of iPSCs into cardiomyocytes (CMs) is a relatively well-established methodology (Lian et al., 2013), allowing iPSC-derived CMs to be used in many different applications. These iPSC-CMs share some important characteristics with adult CMs: In terms of gene expression, iPSC-CMs show a pattern that is consistent with adult CMs (Kattman et al., 2011; Burridge et al., 2014; Bedada et al., 2016). Functionally, iPSC-CMs display most major types of ion current seen in adult CMs, including the fast inward sodium current (I_Na_), the transient outward potassium current (I_to_), the L- and T-type calcium currents (I_CaL_ and I_CaT_), the rapid and slowly activating delayed rectifier potassium currents (I_Kr_ and I_Ks_), and the hyperpolarization-activated pacemaker current (I_f_) (Ma et al., 2011; Liang et al., 2013; Knollmann, 2013). In addition, iPSC-CMs can be created with genetic mutations that are presented in inherited cardiovascular diseases such as long QT syndrome (Moretti et al., 2010; Itzhaki et al., 2011; Yazawa et al., 2011; Egashira et al., 2012; Terrenoire et al., 2013), catecholaminergic polymorphic ventricular tachycardia (Fatima et al., 2011; Itzhaki et al., 2012; Jung et al., 2012; Kujala et al., 2012), and arrhythmogenic right ventricular cardiomyopathy (Ma et al., 2013). Using iPSC-CMs to investigate these mutations can provide crucial insights into cellular arrhythmia mechanisms and the genotype-phenotype correlation of cardiovascular diseases.

In drug screening and discovery, iPSC-CMs can be used to evaluate proarrhythmic risk. Here, iPSC-CMs can be used as *in vitro* models that closely resemble human physiology and patient-specific conditions (Ebert et al., 2012; Mathur et al., 2015; Avior et al., 2016). Recently, such *in vitro* studies have become more important for drug evaluation (Friedrichs et al., 2005; Pugsley, 2005; Lindgren et al., 2008; Giorgi et al., 2010) and the use of iPSC-CMs in drug safety pipelines has been proposed by the Food and Drug Administration (FDA)-led “Comprehensive *in vitro* Proarrhythmia Assay” (CiPA) initiative (Sager et al., 2014; Ando et al., 2017). As part of CiPA it is intended that iPSC-CMs act as a check on mathematical model predictions of pro-arrhythmic risk.

However, some care needs to be taken when interpreting the results of experiments on iPSC-CMs, as many differences between iPSC-CMs and adult CMs still exist. For example, iPSC-CMs have a smaller average cell size (Polak and Fijorek, 2012), lack T-tubules (Lieu et al., 2009) and have lower contractile force (Rodriguez et al., 2014). Their calcium handling machinery is underdeveloped, including changes to calcium-induced calcium release, buffering in the sarcoplasmic reticulum and recycling of calcium by SERCA (Sedan and Binah, 2011; Blazeski et al., 2012), although this is still under debate (Hwang et al., 2015). The expression levels of some ion channel genes also show some important differences. Unlike adult CMs, iPSC-CMs have little I_K1_ current (van den Heuvel et al., 2014), and a prominent I_f_ current (Knollmann, 2013; Keung et al., 2014). These different current characteristics of iPSC-CMs give rise to a relatively positive diastolic potential and slower upstroke velocity compared with adult CMs. The need to further understand these sub-cellular differences, to translate findings in iPSC-CMs to adult myocytes, and to understand how they relate to cell and tissue-level effects, has driven researchers to develop computational models of iPSC-CMs (Paci et al., 2013, 2015).

Each iPSC-CM cell line is developed from a donor with a particular genetic background, using a specific set of protocols from differentiation to maturation. Besides the differences in iPSC-CM and adult-CM electrophysiology, differences between iPSC-CM cell lines have also been shown (Okano et al., 2013; Priori et al., 2013; Moran et al., 2014; Du et al., 2015). Cell-to-cell variability of ion current characteristics within a single line of iPSC-CMs was also observed (López-Redondo et al., 2016) which, as in adult CMs, can have strong implications for our understanding of cell electrophysiology and prediction of drug effects (Mirams et al., 2016).

To use and trust iPSC-CMs as an *in vitro* model for drug screening and disease modeling, it is crucial to evaluate the differences between cell lines and the intra-cell line variability, and to understand how these differences impact experimental outcomes (Karakikes et al., 2015; Del Álamo et al., 2016). Computational modeling can be used to understand and to quantify this intra- and inter-cell line variability, and to gain mechanistic insights into iPSC-CM electrophysiology.

But how detailed does such modeling work need to be? Can a model based on one cell line be used to make inferences about another? How much, and what type of experimental data is needed to tailor a model to a new cell type, or even an individual cell?

In this exploratory study, we compared electrophysiological characteristics of the Cor.4U iPSC-CM cell line (Axiogenesis AG, Germany) to a model by Paci et al. (2013), based on the Ma et al. (2011) studies of an iPSC-CM cell line from Cellular Dynamics International (CDI), iCell. First, we measured the maximum conductances of sodium, calcium and lumped outward currents in individual Cor.4U cells, and by comparing this to model predictions we attempted to infer the maximum conductances of the individual ionic currents. We focused on the maximum conductances of I_Na_, I_CaL_, I_Ks_, I_NaCa_. These maximum conductances were then used to tailor the Paci et al. (2013) model to create cell-specific models of 22 different Cor.4U cells. Using these tailored models to simulate APs, we found a variety of AP waveforms exhibiting a high level of variability similar to that found in real iPSC-CMs. We then optically measured action potential durations (APDs) in iPSC-CM cultures under both control and drug-applied conditions, and found that—in most cases—tailored models predicted the resulting changes better than the original model. This suggests that the ion current composition differs between cell lines, and highlights the need to tailor *in silico* models to different cell lines to interpret drug-induced alterations to their electrophysiology. Our results also show that even a relatively simple approach, in which only the maximum conductances are considered with limited experimental data, can already provide useful information in this regard, but that more intricate methods will be needed to characterize differences in outward currents between iPSC-CM cell lines.

## 2. Methods

### 2.1. Current measurements in Cor.4U cells

Sodium, calcium, and lumped outward currents were measured in Cor.4U cells in the whole-cell patch clamp configuration using the Nanion SyncroPatch 96 platform (Nanion Technologies GmbH, Germany). Sodium and lumped outward currents were measured using an intracellular solution containing (in mM) 50 KCl, 60 KF, 10 NaCl, 10 HEPES, and 20 EGTA (pH: 7.2), and a bath solution containing (in mM) 150 NaCl, 4 KCl, 1 MgCl_2_, 1.2 CaCl_2_, 10 HEPES, and 5 glucose (pH: 7.4). Calcium current recordings were made using an intracellular solution containing (in mM) 50 CsCl, 60 CsF, 10 TEA-Cl (a potassium current blocker), 5 HEPES, 10 EGTA, 4 Na2-ATP, 0.1 Na-GTP, and 0.1 cAMP (pH: 7.2) and a bath solution containing (in mM) 130 NMDG, 10 BaCl_2_, 4 CsCl_2_, 1 MgCl_2_, 2 CaCl_2_, 10 HEPES, and 5 glucose (pH: 7.4). All currents were recorded at room temperature.

For the sodium current measurements, cells were held at −80 mV and then stepped to potentials ranging from −60 to 60 mV with 10 mV increments, before returning to the holding potential. The step duration was 20 ms and the interval between steps was 5 s. The calcium current experiments used a similar protocol, but with 200 ms steps from −40 to 40 mV. Outward current was measured with 500 ms steps from −40 to 50 mV, with a 10 s interval between steps. All three protocols are shown in Supplementary Figure S1.

For the outward current experiments, we fitted directly to the experimental current traces (see section 2.5), and so leak correction was applied using *I*_*leak*_ = *V*/*R*_*leak*_ where *R*_*leak*_ was the leak resistance estimated at the holding potential. Capacitance artifacts were filtered out by omitting the first 10 ms after each change in potential (see e.g., Ogden and Stanfield, 1994).

### 2.2. Patch clamp AP measurements in iPSC-CMs

Action potentials in iCell iPSC-CMs (CDI, USA) plated on coverslips were measured in whole-cell patch clamp configuration using a HEKA amplifier (EPC 10 USB Triple, HEKA Elektronik, Germany). Recordings were made using a pipette solution containing (in mM) 10 NaCl, 125 KCl, 1 MgCl_2_, 10 HEPES, 0.1 Na_3_GTP, 5 Mg-ATP, 5 EGTA (pH 7.2) and a bath solution containing (in mM) 150 NaCl, 4 KCl, 1.2 CaCl_2_, 1 MgCl_2_, 10 HEPES (pH 7.4). Cells were stimulated at a frequency of 1.0 Hz, for at least 50 cycles before recording.

### 2.3. Optical mapping AP measurements in Cor.4U cultures

Action potentials were recorded from Cor.4U cultures with optical mapping using the CellOPTIQ electrophysiology platform (Clyde Biosciences Ltd). Cells were incubated in serum-free media at 35 ± 2°C, and transiently loaded with voltage sensitive fluorescent dye di-4-ANEPPS (20 μL of stock solution 27 mM in ethanol; University of Connecticut Health Center). The loaded dye was then excited with a peak wavelength 470 nm LED, and the emitted fluorescence from the Cor.4U iPSC-CMs was recorded at a sample frequency of 10 kHz. Measurements were performed before and after addition of Dofetilide, Quinidine, Sotalol and Verapamil at the concentrations shown in Table 1. Paracetamol was applied as a negative control.

**Table 1:** Summary of the applied reference drugs which are a variety of multi-channel blockers, including the IC50 values for the corresponding ion channels, and the applied drug concentration (*x*).

**IC50 [μM]**	**Dofetilide**	**Quinidine**	**Sotalol**	**Verapamil**
I_Kr_	0.0052^a^	0.3^b^	111.4^d^	0.25^d^
I_Na_	147.9^a^	16.6^c^	7013.9^d^	32.5^d^
I_CaL_	26.7^a^	15.6^c^	193.3^d^	0.2^d^
I_Ks_	415.8^a^	No significant effect	No significant effect	No significant effect
*x* [μM]	0.03, 0.1, 0.3, 1.0	0.01, 0.1, 1.0, 10	0.3, 3.0, 30, 300	0.01, 0.1, 1.0, 10

The IC50 data are from

a , Obejero-Paz et al. (2015);

b , Po et al. (1999);

c , Mirams et al. (2011); and

d *, Kramer et al. (2013)*.

A semi-automatic data analysis method based in Wang et al. (2015) was employed to normalize the data. In short, heuristics were used to form an initial estimate of the start and end time of the AP. The region just before the estimated upstroke was used to determine *V*_normalized_ = 0, while the 95th percentile of the data during the (estimated) AP was used as *V*_normalized_ = 1. We then calculated the final APD_90_ and APD_50_ from this normalized signal.

### 2.4. Simulated experiments

Simulations of the patch clamp protocols were carried out using the model by Paci et al. (2013). Initial intracellular and extracellular ion concentrations were set to the values used in the experiments. For the I_Na_, I_CaL_, and I_outward_ voltage clamp experiments, concentrations were clamped (corresponding to the buffering effects of the pipette), but for AP simulations concentrations were allowed to vary following model equations. The temperature parameter in the model, which affects reversal potentials as well as I_CaL_ permeability and I_Kr_, I_NaK_, and I_NaCa_ kinetics, was set to 25°C (298 K) to match the experimental temperature. Simulations were run using Myokit (Clerx et al., 2016), with CVODE (Hindmarsh et al., 2005) set to the default tolerance settings of abs_tol = 10^−6^ and rel_tol = 10^−4^. Model code was imported from a CellML (Cuellar et al., 2003) file downloaded from the Physiome model repository (Yu et al., 2011). Numerical integration was carried out using NumPy/SciPy (Jones et al., 2001). All codes and data are freely available from https://gitlab.com/MichaelClerx/tailored-ipsc-models.

### 2.5. Estimating maximum conductances of individual ion currents

The maximum conductance of I_Na_ was estimated by scaling the I_Na_ conductance in the Paci model to match the peak current recorded experimentally with the sodium protocol (*n* = 35 cells), based on the assumption that the peak current is composed of I_Na_ alone. We tested this assumption by running a simulated experiment, where we observed that I_Na_ alone would reach 1.01 × the initial inward deflection after each voltage step. So the peak is almost entirely due to sodium and only decreased slightly by the presence of other currents. Similarly, the recordings made with the calcium protocol (*n* = 25 cells) were used to directly infer the maximum conductance of I_CaL_.

To estimate the conductances of the remaining major currents, we used the recordings made with the outward-current protocol (*n* = 22 cells). Using the iPSC model by Paci et al. (2013) we simulated the response to this protocol of I_Na_, I_CaL_, I_K1_, I_Kr_, I_Ks_, I_to_, I_f_, and I_NaCa_ (see Supplementary Figure S2). We then tried to find a weighted sum of these simulated currents that could replicate the measured signal. This was done by minimizing the sum of square errors between measured and simulated current during the voltage steps, using the optimization method CMA-ES (Hansen, 2006). The procedure was repeated for each of the 22 measured cells, resulting in a unique set of scaling factors per cell.

While this protocol was intended to find values for only the outward currents (such as I_Kr_, I_Ks_, and I_to_) we chose to vary the inward currents I_Na_ and I_CaL_ in the optimization to reduce the risk that any inward currents in the signal would erroneously be attributed to the outward currents (see e.g., Sarkar and Sobie, 2010). Note that we do not use these fitted I_Na_ and I_CaL_ conductances because we fit these from other dedicated experiments; they were included here just to yield more accurate outward current fits. After finding that the most prominent outward currents were I_Ks_ and I_NaCa_ (see section 3.2), we ran a second optimization with only I_Ks_ and I_NaCa_: little change was observed, but we expect that fitting outward currents together with I_Na_ and I_CaL_ is likely to yield slightly more accurate results.

Convergence of the optimization results was verified by repeating the process 10 times, using different random seeds for each run. We found that the L^2^ norm of the difference between the first and repeated scaling factor vectors was smaller than 10^−5^ for all 10 random starting points. To further verify the identifiability of the problem, we performed the same analysis on synthetic data (with synthetic noise), and were able to successfully infer the conductance scaling factors (see Supplementary Figure S4 and Supplementary Table S1). We note that this analysis assumes the kinetics of the currents have low model discrepancy, i.e., reflect the kinetics of the real currents well.

Finally, to quantify the contribution of each current to the total outward current, we defined a contribution score *c*_*i*_ for each current *I*_*i*_ as:

(1) $$\begin{array}{l} c_{i}=\frac{\left|I_{\text{final},i}\right|}{\underset{j}{\sum }|I_{\text{final},j}|} \end{array}$$

where *I*_final, *i*_ was defined as the current measured at the end of the final step of the outward-current protocol. This measure simply gives us a sense of the proportion of outward current that is contributed by each individual component during the end of the 50 mV step, but it is not used in tailoring the models.

### 2.6. Predicting the shape of the AP

Next, the estimated maximum conductances were used to tailor the Paci et al. (2013) model to individual cells from the Cor.4U cell line. A total of 22 model variants were parameterized, corresponding to the 22 cells for which the outward current was measured. Since we found many currents were not discernible in the recorded outward current (see Table 2), we only applied the cell-specific scaling factors for I_Ks_ and I_NaCa_. All tailored models used the same I_Na_ and I_CaL_ scaling factors, found in the inward current experiments which were measured in different cells and hence any covariance could not be accounted for. The remaining currents were left unchanged, as they are necessary for other cellular behavior, such as homeostasis, even though they might not contribute strongly to the recorded outward current. Note that we have used only linear scaling of the conductances, and the current kinetics of the original currents were not altered. Finally, Na^+^, K^+^, and Ca^2+^ evolve in time according to the Paci et al. (2013) model, to mimic the intact cell conditions of our optical mapping experiments.

**Table 2:** The scaling factors (*s*) and the relative contribution (*c*) of individual ion currents to the measured outward current in Cor.4U cells (n = 22).

**Cell**	**I_NaCa_**		**I_Ks_**		**I_Kr_**		**I_K1_**		**I_f_**	
	***s***	***c***	***s***	***c***	***s***	***c***	***s***	***c***	***s***	***c***
1	3.32	28.5	139	71.2	—	—	—	—	—	—
2	2.08	21.2	129	78.5	—	—	—	—	—	—
3	7.55	50.3	125	49.5	—	—	—	—	—	—
4	21.1	43	467	56.8	—	—	—	—	—	—
5	6.89	29.9	270	70	—	—	—	—	1.18	—
6	9.3	42.4	211	57.4	—	—	—	—	—	—
7	0.951	29.6	37.6	69.9	—	—	—	—	—	—
8	1.54	56.7	19.5	42.8	—	—	0.118	0.000258	—	—
9	1.7	35.2	52	64.3	—	—	—	—	—	—
10	1.67	98.6	—	—	—	—	0.104	0.000362	—	—
11	0.38	16.7	31.5	82.5	—	—	—	—	—	—
12	0.696	38.2	18.6	60.9	—	—	—	—	—	—
13	3.05	25	153	74.7	—	—	—	—	—	—
14	1.28	14.7	125	84.9	—	—	—	—	—	—
15	1.19	34.1	38.3	65.3	—	—	—	—	—	—
16	3.02	51.4	47.3	48	—	—	—	—	—	—
17	2.35	59	27	40.4	—	—	—	—	—	—
18	4.03	32.7	138	66.9	—	—	—	—	—	—
19	5.74	73.4	28.9	22	1.84	4.5	0.513	0.000388	—	—
20	2.35	80.5	9.18	18.8	—	—	1.18	0.0024	—	—
21	3.77	40.2	93.7	59.6	—	—	—	—	—	—
22	13.9	44.6	288	55.1	—	—	—	—	—	—

*Values lower than 10^−10^ are shown as dashes (—). The scaling factors are taken with respect to the maximum conductance found in the original Paci et al. (2013) model. I_to_ values were lower than 10^−10^ for all cells. Because of the many low values for I_to_, I_Kr_, I_K1_, and I_f_, only the values for I_NaCa_ and I_Ks_ were used to create the tailored models*.

These tailored models were then used to simulate baseline APs, as well as APs with drug perturbation. The effects of drugs on ion current maximum conductances were modeled using the Hill equation (Hill, 1910; Weiss, 1997).

(2) $$\begin{array}{l} f\left(x\right)=\frac{1}{1+{\left(x/\text{IC}50\right)}^{h}} \end{array}$$

where *x* denotes the concentration of the applied drug, IC50 is the inhibitory concentration 50% value, *h* is the Hill coefficient, and *f*(*x*) is a scaling factor for the maximum conductance that varies from 0 (full block) to 1 (no block).

For each cell and each drug, a model was created where the maximum conductances of the ion currents were scaled according to Equation (2) using the IC50 values from Table 1 and a Hill coefficient of 1.0. For comparison, the same scaling factors were applied to an original model with the untailored conductance values from Paci et al. (2013).

In our optical mapping experiments, cells formed a spontaneously-beating and electrotonically-coupled monolayer. However, in this preparation not all cells beat at their spontaneous rates. Most cells will fire an AP when triggered by an activation wave from their neighbors rather than spontaneously, and a relatively small region of (by definition) faster spontaneously-beating cells sets the pacing rate for the entire monolayer. Therefore, to mimic this effect, we paced the cells at the mean rates observed in the optical mapping experiments, for a given compound, to account for any AP rate dependency. We used the cycle lengths of 1.375 s for Dofetilide, 1.176 s for Quinidine, 0.933 s for Sotalol, 0.905 s for Verapamil and 1.0 s for Paracetamol and all other experiments. To allow direct comparison with the optical mapping data, the simulated AP was normalized using the same algorithm (see section 2.3).

## 3. Results

### 3.1. I_Na_ and I_CaL_ in Cor.4U cells

**Figure 1:**
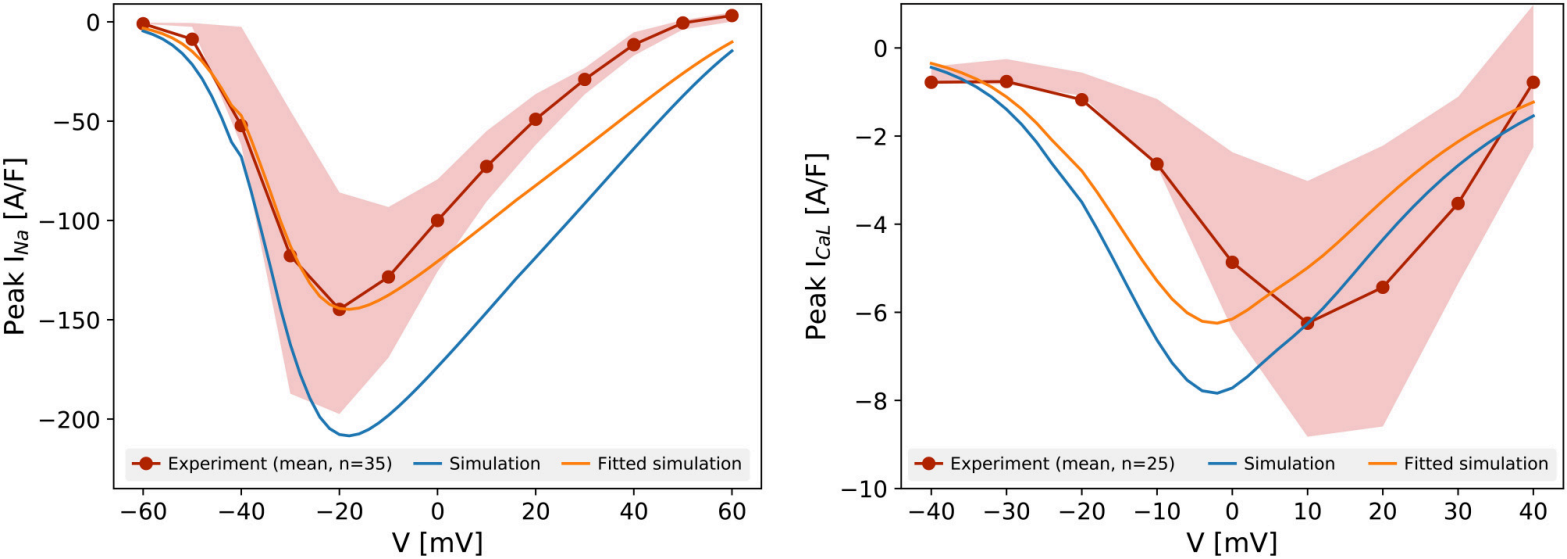
Current-voltage relationship for I_Na_ **(left)** and I_CaL_ **(right)**. The red lines represent the mean peak current measured experimentally in 35 (I_Na_) and 25 cells (I_CaL_), and the shaded areas show the 25th and 75th percentiles of the experimental data. The peak-current voltage relation simulated with the unaltered Paci et al. (2013) model for the same protocol is shown in blue. The orange lines show the simulated results after scaling to match the maximum current.

Figure 1 shows the peak current-voltage relationships for I_Na_ and I_CaL_, measured in Cor.4U cells. The mean peak current in 35 cells (I_Na_) and 25 cells (I_CaL_) is plotted, as are the 25th and 75th percentiles. Compared to the prediction of the original Paci et al. (2013) model (created from iCell iPSC-CM data), the experimental data show a lower amplitude of both currents in Cor.4U cells. We had to scale by a factor 0.69 to match the mean peak I_Na_, and 0.80 to match the mean peak I_CaL_ recordings. The simulated I_Na_ peaked at the same potential as the experimental data, suggesting the activation kinetics of I_Na_ in iCell and Cor.4U cell lines are similar. The simulated I_CaL_ kinetics followed the Paci et al. (2013) model, and were left-shifted relative to experimental data. Further experiments established that this shift was due to a right-shift in the experimental IV curve due to Ba^2+^ being present in the I_CaL_ voltage clamp experiment bath solution (see Supplementary Figure S3), hence we do not adjust the kinetic terms and tailor only the maximum conductance.

### 3.2. The outward protocol strongly elicits I_Ks_

**Figure 2:**
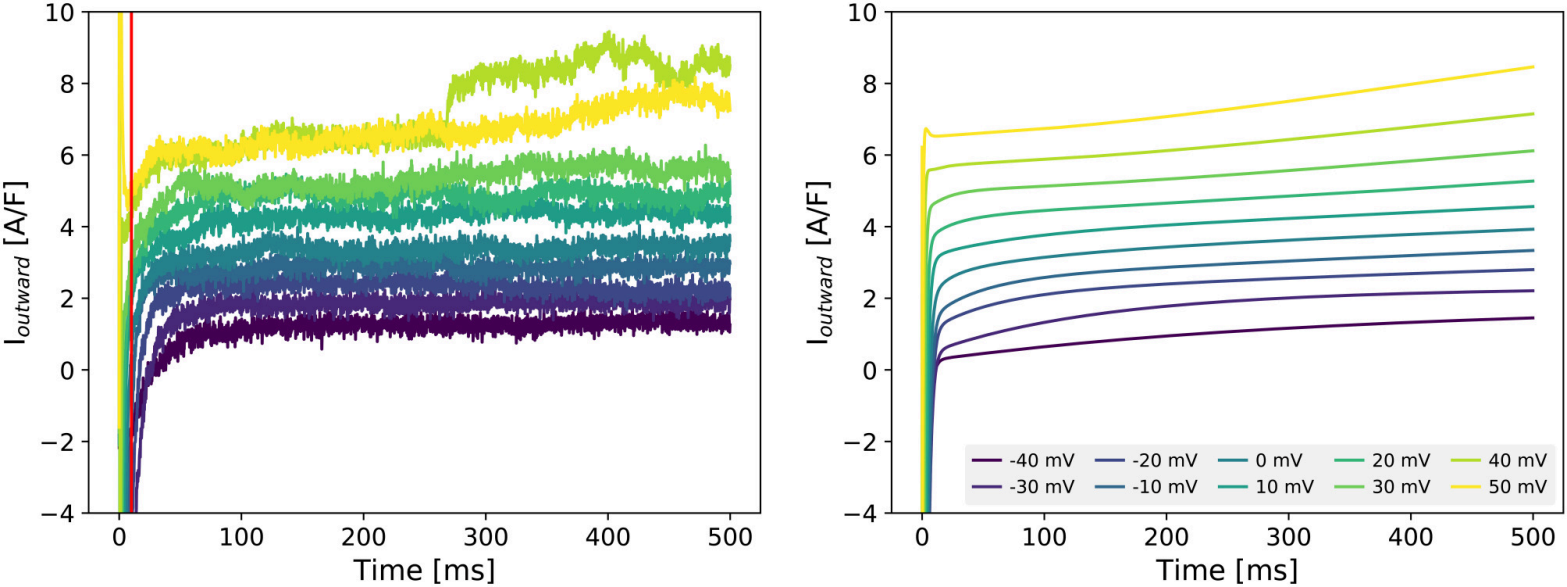
**Left:** Experimentally measured outward current in cell 19 during the outward protocol. Data to the left of the vertical red was omitted to remove capacitance artifacts. **Right:** Simulated transmembrane current during the same protocol, as set during the optimization process for cell 19. Note that this figure includes *all* scaling factors set by the optimization routine (see section 2.5), including ones not included in the final tailored models (such as I_Na_ and I_Kr_). More examples of fits are shown in Supplementary Figure S5.

Figure 2 (left panel) shows the current measured with the outward-current protocol in a single Cor.4U cell. To analyse the composition of this current, we simulated the same protocol, and looked for a sum of scaled transmembrane currents from the Paci et al. (2013) model that gave a similar result (see section 2.5). We repeated this process for each of the 22 cells with measured outward current, and obtained the scaling factors *s* for each cell and current shown in Table 2. Note that the scaling factors *s* are *relative* to the original Paci et al. (2013) model. In many cases, the optimization routine indicated that the kinetic profile of certain currents was not discernible in the measured outward current. This is indicated in the table with a dash (—) for any scaling factor smaller than 10^-10^. After seeing these results, as a comparison, we also tried fitting by varying only I_Ks_ and I_NaCa_ (and using the scaling factors for I_Na_ and I_CaL_ determined previously), and the results are similar (see Supplementary Table S2).

For most cells, we found that the measured responses differed greatly, in both the shape and size of the currents, from the original model predictions (see Supplementary Figure S4), leading to a poor quality of fit (see Supplementary Figure S5). As a result, the best reconstructions of the simulated current relied almost entirely on a greatly amplified I_Ks_ current, along with strong I_NaCa_, while other currents such as I_Kr_ and I_f_ were notably absent. Based on this, we might assume that I_Ks_ and I_NaCa_ are more strongly expressed in Cor.4U cells than in the iCell cells the Paci et al. (2013) model was based on. As an initial verification of these findings, we repeated some outward current measurements in the presence of Chromanol (an I_Ks_ blocker), see Supplementary Figure S6 for an example where I_Ks_ is indeed significant. The near-zero contributions of other currents (e.g., I_Kr_) does not imply that these currents are completely absent in Cor.4U cells, but instead suggests that the currents *as simulated from the model* could not be found in our recordings using the specified patch clamp protocol. This is a strong hint that changes to the kinetics of the currents will be required to accurately simulate the ion currents in Cor.4U cells at this temperature using the model by Paci et al. (2013). Such a mismatch in kinetics would also explain the large remaining errors between measurements and fit seen in Supplementary Figure S5, causing other currents, such as I_Kr_, to be fitted as absent. This is discussed further in section 4.4.

### 3.3. Tailored models

We then created tailored models by modifying the original Paci et al. (2013) model in two ways: First, we scaled the maximum conductances of I_Na_ and I_CaL_ by a factor 0.69 and 0.8 respectively, to match the averaged data from the inward current experiments. We then further modified this model to create 22 tailored models based on the 22 cells in which outward current was measured, by applying the I_Ks_ and I_NaCa_ scaling factors from Table 2.

### 3.4. Variability in ioutward predicts variability in AP

Significant variability in the outward currents was observed among the Cor.4U cells. This can be seen from the scaling factors in Table 2, but it is also evident when directly inspecting the currents measured from different cells (see Supplementary Figure S5) or when looking at peak I_outward_ (see Supplementary Figure S7).

**Figure 3:**
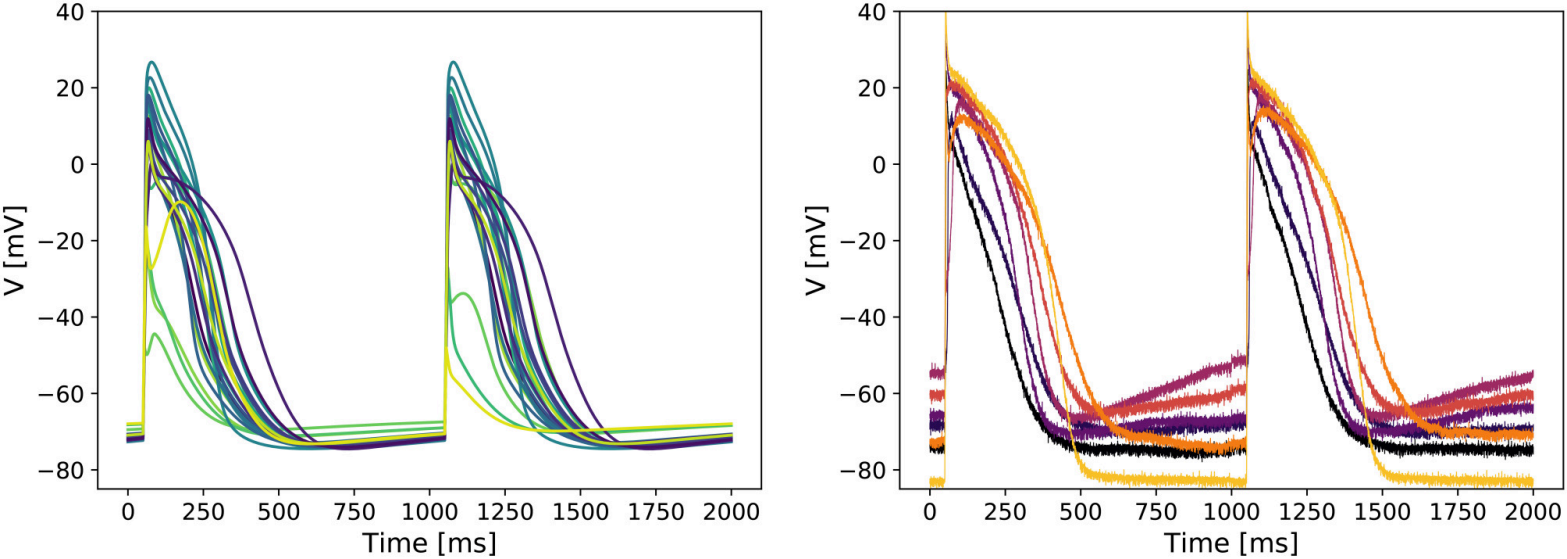
Predicted variability in the tailored action potential models is similar to inter-cell variability in a batch of iPSC-CMs. **Left:** Simulated APs from the 22 tailored cell-specific Cor.4U models exhibit a variety of AP waveforms. **Right:** Experimentally measured APs in seven individual iCell iPSC-CMs also show significant variability.

Figure 3 (left panel) shows APs simulated with the tailored models. A wide variety of APs could be seen, with some models showing a spike-and-dome waveform, some showing a more triangular waveform, and with a varying slope in resting potential (leading to different degrees of auto-excitation). Some models also show beat-to-beat alternans, or fail to completely depolarize. The corresponding contribution of the major currents throughout the APs are also shown in Supplementary Figure S8.

Recordings of APs in single iPSC-CMs show a similar variety of AP waveforms. Figure 2 (right panel) shows APs measured in 7 different iCell iPSC-CMs. Again, various waveform morphologies (roughly corresponding to atrial, ventricular and sinoatrial node APs) and differing levels of auto-excitability can be distinguished. Whilst these recordings are for a different cell line than our tailored models, the inter-cell variability in channel expression within a batch of iPSC-CMs has not been observed to be markedly different between cell lines (see e.g., the relative size of the “error bars” in Figure 2 of Blinova et al., 2017).

### 3.5. Tailored models improve predictions of APD

**Figure 4:**
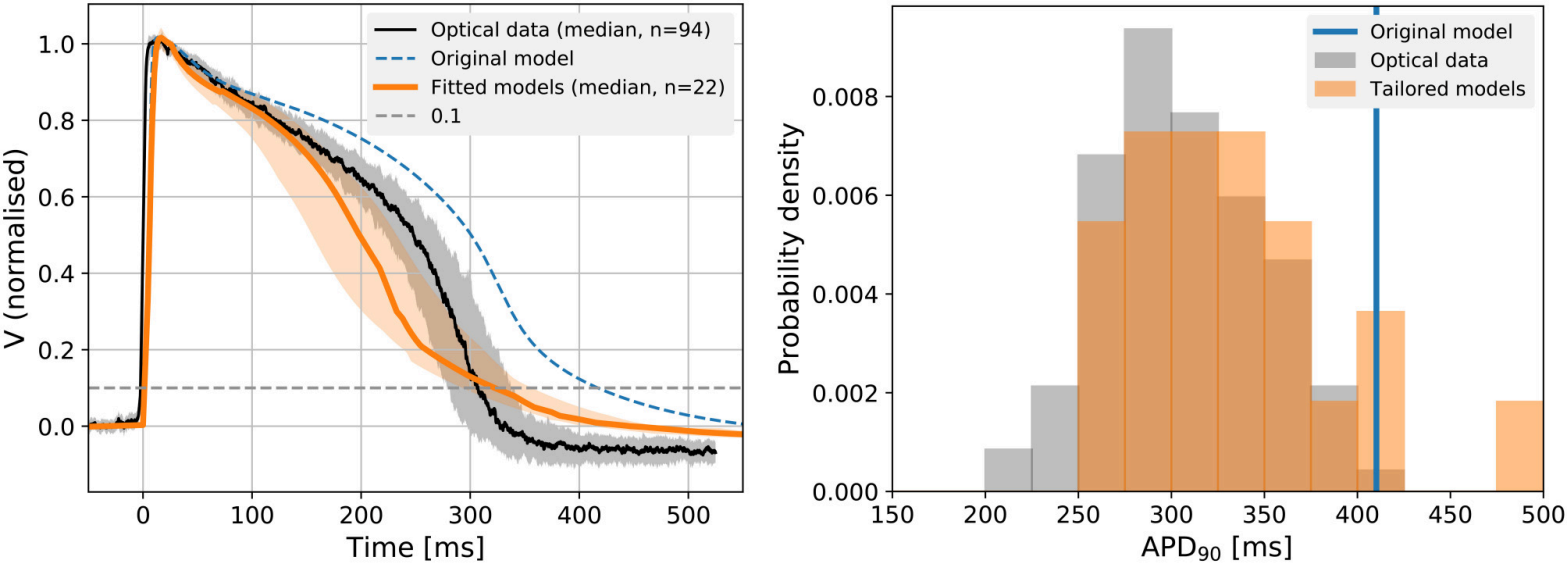
**Left:** The predicted 1 Hz steady pacing APs from the individual cell optimized models (orange), the original Paci model (dashed blue), and optical mapping measurements (black) in the control conditions. We show the median and 25th and 75th percentiles of the optical mapping (gray) and action potential models (orange). All data shown are normalized (see section 2.3). For models that exhibited strong alternans (i.e., where only every second AP showed a spike-and-dome morphology) the longer of the two APs was used. **Right:** A histogram of APD_90_ in the fitted models and optical mapping control (drug free) experiments. As we might expect, there is more variation in APD in the individual-cell tailored action potential models than the electrotonically-coupled tissue measurements, but the distribution is centred appropriately.

Figure 4 (left panel) shows the median of all simulated traces as shown in Figure 3, along with the 25th and 75th percentiles. The optically recorded APs from the Cor.4U cells were plotted on the same graph (the median shown as black line and the 25th and 75th percentiles shown as gray shading). Due to the increased outward current, the tailored models exhibit a shorter APD than the original model, that matches the measured APDs more closely in the early and late repolarization phase. A histogram of APDs in measured and simulated cells is shown in Figure 4 (right panel), with the blue line representing the result from the original model. A similar histogram for APD_50_ is shown in Supplementary Figure S9.

### 3.6. Tailored models can give better prediction of drug block effects

**Figure 5:**
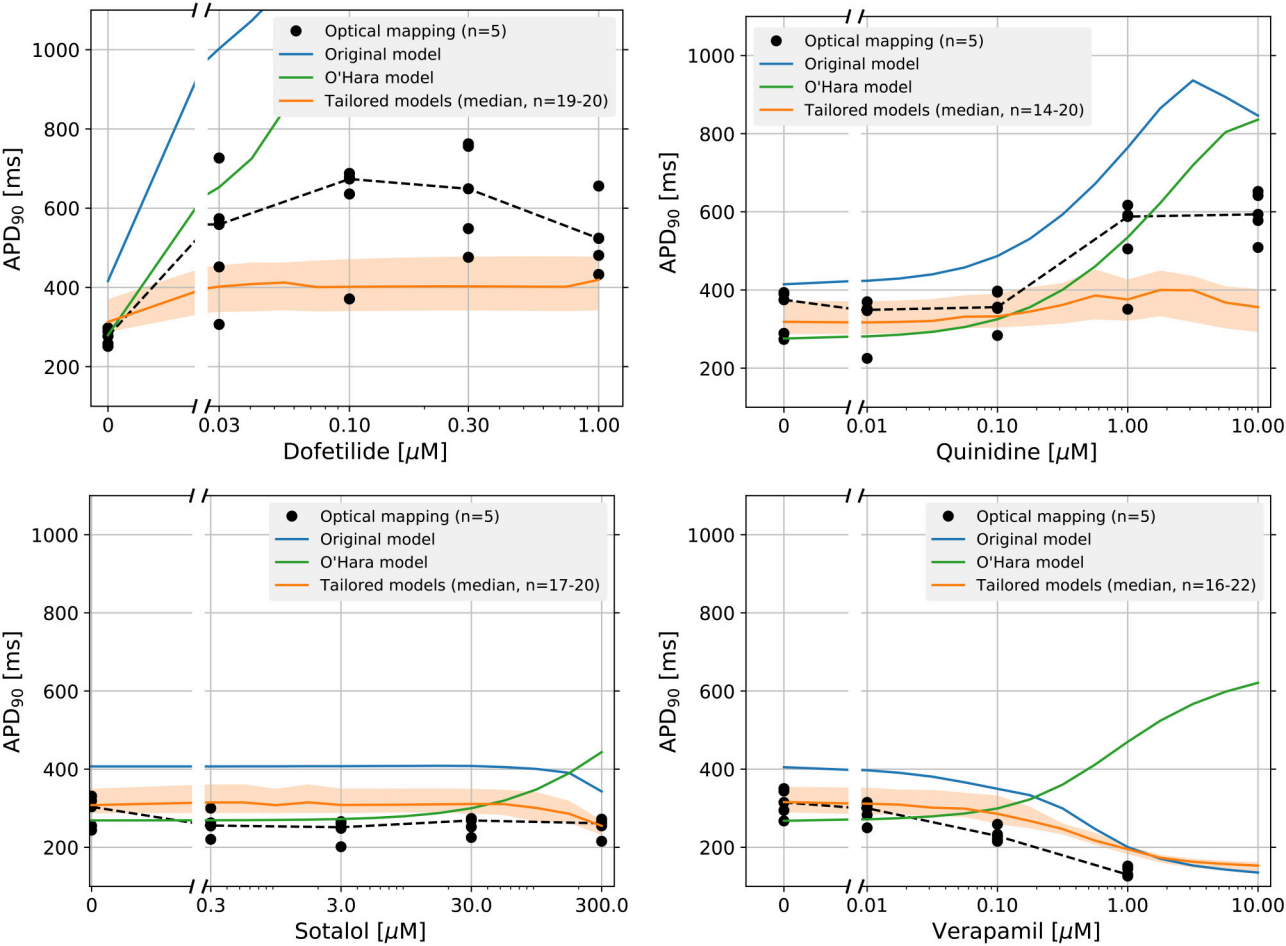
Dose-response curves of the APD_90_ for four drugs: Dofetilide, Quinidine, Sotalol, and Verapamil. The individual optical mapping measurements are shown as black dots, with the median shown as a dotted black line. Predicted responses from the original model are shown in blue, and the tailored model predictions are shown in orange (solid line is median and shaded region indicates 25th–75th percentiles. Models (tailored or original) that exhibited strong alternans (i.e., where only every second AP showed a spike-and-dome morphology) were omitted from the figure. Because this caused the number of predictions in the tailored model distribution to vary, the minimum and maximum number of predictions per drug is shown as *n* = *minimum* − *maximum*. At higher concentrations, Dofetilide block causes repolarization failure in both the original model and the O'Hara model.

Figure 5 shows the dose-response curves of the APD_90_ of four drugs, measured experimentally and simulated using the original and tailored models. Equivalent results using the of the APD_50_ are shown in Supplementary Figure S9. Results for the control drug paracetamol are shown in Supplementary Figure S10. For all four drugs tested, although not fitting the experimental data exactly, the tailored models match the measured data more closely than the original model. For Dofetilide in particular, the tailored models show a realistically smaller increase in APD than the original model, which shows alternans and then repolarization failure at higher drug concentrations. For Quinidine, although the tailored models do not fit better at the highest concentration, we improve the predictions at lower concentrations.

Predictions made with the adult-CM model by O'Hara et al. (2011) are shown for comparison. Note how the adult CM model predicts APD prolongation with Verapamil, whereas both the tailored and original iPSC-CM models accurately predict the shortening that is observed in iPSC-CM optical mapping. Such qualitative differences highlight the need for models specific to iPSC-CMs to interpret experimental findings in these cells.

## 4. Discussion

Pre-clinical studies with iPSC-CMs can be used to evaluate proarrhythmic risk of compounds at the early drug discovery and development phase for compound optimization, and these experimental results can directly contribute to the design of safe first-in-human doses. Ideally, for reliable risk identification and translation, the electrophysiology of iPSC-CMs should accurately reflect that of adult cardiomyocytes. Yet the characteristics of iPSC-CMs are influenced by donor genetic background as well as differentiation and maturation protocols, and so differences between iPSC-CM cell lines may be expected, as well as differences from adult CMs. Mathematical models of the cellular AP can be used to gain mechanistic insight into such differences and to build a quantitative translational framework between iPSC-CMs and human adult CMs.

In this study we compared novel measurements in Cor.4U iPSC-CMs with predictions from a model based on the iCell cells. We found a decrease in I_Na_ and I_CaL_ current densities, but a large increase in I_Ks_ and more modest increases in I_NaCa_. Using the simple method of scaling maximum conductances—without altering ion current *kinetics*—we created models tailored to individual iPSC-CMs. The obtained fits were not optimal, which suggests that the ion current kinetics in the iCell-cell based model by Paci et al. (2013) do not closely match those in Cor.4U cells. However, like real iPSC-CMs, these tailored models show differences in AP from cell to cell, with AP waveforms broadly similar to ventricular, atrial and sinoatrial-node APs. The predicted single-cell APD_90_ was shorter in tailored models than in the original model, and showed a better match with optical mapping measurements in electrotonically-coupled iPSC-CM cultures. The effects of Dofetilide, Quinidine, Sotalol and Verapamil on APD were simulated, and again the tailored models provided a closer fit. These results show that there are important electrophysiological differences between iPSC-CM cell lines, but that relatively simple adjustments to computational iPSC-CM models can already partially accommodate them. This has important implications for the suggested drug-screening workflows: one should really combine both iPSC-CM measurements and computational modeling of iPSC-CM for better interpretation of the iPSC-CM data in terms of its variability and translational power.

### 4.1. Cell-line differences in ion current densities

We obtained maximum conductance values for the inward currents I_Na_ and I_CaL_ that are lower than suggested by the Paci et al. (2013) model based on iCell cells, while I_Ks_ and I_NaCa_ were increased in most Cor.4U cells. The slight reduction in I_CaL_ and increase in I_Ks_ suggests a decrease in APD. This was borne out by the AP simulations, and was consistent with our optical mapping measurements which showed shorter APDs compared to the AP simulated by the original Paci model. Our simulations displayed a similar degree of AP variability to the experimental iPSC recordings, but larger variability than the optical mapping measurements. Both findings are consistent given that electrotonic coupling of cells (present in the optical mapping experiments) reduces variability.

A potential explanation of the large I_Ks_ current is suggested by Lei et al. (2017). It shows that both the KCNQ1 and KCNE1 (subunits of the channel carrying I_Ks_) were present in our Cor.4U cells, however, KCNE1 was not as well expressed in iCells. The difference in KCNE1 expression could lead to the observed larger I_Ks_ currents in the Cor.4U cells compared to iCells, and hence a shorter APD and less prolongation under I_Kr_ blockers, which is in agreement with Blinova et al. (2017). Our observation is supported by Silva and Rudy (2005) who found that native I_Ks_ (from channels comprised of both KCNQ1 and KCNE1) activates more than with KCNQ1 only.

### 4.2. Cell-to-cell differences in iPSC-CMs

iPSC cardiomyocytes, from the same donor and differentiated/matured in the same way, can display vastly different AP waveforms, reminiscent of those of ventricular, atrial, and sinoatrial-node cells. Our tailored models, created by varying the maximum conductances of I_NaCa_ and I_Ks_, showed a similar model-to-model (cell-to-cell) variety in style of generated APs. This shows that variation in genetic expression, which correlates directly with maximum conductance (Schulz et al., 2006), could be enough to explain the different AP waveforms observed in iPSC-CMs. However, it does not preclude other explanations, and it is possible the APs could take on a more distinct shape if differences in ion channel kinetics were also included. As discussed in a recent white paper, the inclusion of cell-cell variability, as well as variability between cell lines is an important research area (Johnstone et al., 2016).

### 4.3. Predictions of drug action

The sharp increase in I_Ks_ seen in our Cor.4U tailored models suggests Cor.4U cells have a stronger reliance on I_Ks_ as a repolarizing force, and will therefore be less likely to show AP prolongation when treated with I_Kr_ blocking drugs (see, e.g., the Figure 5 of Blinova et al., 2017, which shows, for 8 out of 12 drugs with comparable concentrations and prolongation in iCells, the Cor.4U cells have a smaller APD prolongation than the iCells). Consistent with this suggestion, simulations of treatment with the potent I_Kr_ blocker Dofetilide showed only a modest increase in APD at concentrations that caused the iCell-cell based model to display excessive AP prolongation resulting in alternans. Treatment with Quinidine, a less potent I_Kr_ blocker, showed similar results. The modest APD increase predicted by the tailored models underestimated the APD prolongation observed in the data, suggesting the role of I_Kr_ as a repolarizing force was underestimated in these models. More refined experiments will need to be conducted to separate the outward currents and to better estimate I_Kr_ conductance. Application of Verapamil, which blocks I_CaL_ as well as I_Kr_, had a smaller effect in our tailored models than in the original model, which is consistent with the lowered levels of I_Kr_ and I_CaL_.

The strong I_Ks_-reliance we observed may be problematic when using these iPSC-CMs as models for ventricular myocytes, where I_Ks_ only plays a major part when other repolarizing currents are blocked or in the presence of sympathetic stimulation (Jost et al., 2005).

### 4.4. Limitations and future work

This study showed the need to build cell-line or even cell-specific models for iPSC-CMs, and this work serves as a pilot attempt for such an approach. However, a refined study with additional experiments will be needed to improve the tailored models further.

As might be expected, the ion current profiles during voltage steps could not be recreated well using this approach. It is likely that ion channel kinetics also vary between cell lines due to (e.g.,) differences in subunit expression (Lei et al., 2017), although this could also be partly due to the difference of temperature, and that the model we used does not accurately capture the kinetics of ion currents in Cor.4U cells. Channel kinetics play an important role in the contribution of a current to the different phases of the AP. Modifying the kinetic parameters which characterize the voltage-current relationship for the activation, inactivation, deactivation, etc. of a channel could change both the current and the AP, and would influence responses to drugs. Varying the kinetic parameters would also alter the conductances we estimated by fitting the outward current. Further tailoring the models to include refitted kinetic parameters may lead to further improvements in predictive power. However, since models of ion channel kinetics contain many parameters, specifically designed experiments (e.g., with channel blockers and/or specialized voltage protocols) will be required to refine these tailored models.

The method of fitting multiple currents to a single experimental recording is a highly useful approach, as it reduces the number of experiments needed to tailor a model. However, due to the limitation of experiments being performed in different cells, we were not able to examine the covariance between the inward and outward currents. Also, since it depends on the number of current conductances to be fitted and the experimental data (e.g., the quality of the data and the actual current shape), one may run into problems of practical identifiability if one tries to refit kinetic parameters here (e.g., multiple combinations of conductance and kinetic parameters that can provide an equally good fit, as in Fink and Noble, 2009). Additional experimental data with refined experimental designs will be needed to identify all parameters; for example, to perform experiments with channel blockers to isolate the contribution of particular currents, or to iteratively refine the models using a dynamic clamp approach (Devenyi et al., 2017).

Variability/noise on drug-ion channel interaction parameters (IC50s) from different labs or repeats of experiments will also impact our simulation predictions. A probabilistic uncertainty quantification framework using the techniques proposed in Elkins et al. (2013), Johnstone et al. (2017) could be used in future to address this.

Our Cor.4U-tailored predictions of both baseline AP and drug responses matched the optical mapping data more closely than the un-tailored model. However, these optical mapping data were gathered from cultures of spontaneously beating electrotonically coupled cells, while our simulations are of paced single iPSC-CMs. Another avenue for future work would be to combine (a representative distribution of) tailored cell-specific models into heterogeneous tissue models (Bowler et al., 2016).

### 4.5. Implications for drug testing

iPSC-CMs have gained significant popularity as an *in vitro* model for drug screening and, as one pillar of the CiPA strategy, are anticipated to become a routine part of the cardiac safety pipeline. It is therefore critical to understand how to interpret the iPSC-CM data variability (intra- and inter-cell line variability) and to translate these data to the adult human situation. Mathematical models are a promising tool to integrate data, gain mechanistic insights and perform this translation.

Our results show that differences between iPSC-CM cell lines can be analyzed and understood using tailored computational models. Furthermore, even models based on relatively simple methods (e.g., scaling maximum conductances) and a limited set of measurements (two inward current and one outward current experiments) can lead to improved predictions of baseline and drug-blocked electrophysiology parameters.

## 5. Conclusions

Using a combination of novel experiments and computational work, we have shown that Cor.4U cells display different ion current densities than the previously characterized model, which is based on iCell data. This included an increased reliance on I_Ks_ for repolarization with an accompanying decreased reliance on I_Kr_. Incorporating these effects in cell-specific models of iPSC-CMs correctly predicted that this would lead to a shortening of the baseline APD and a reduced reaction to I_Kr_-blocking drugs. These predictions were confirmed in optical mapping experiments with reference drugs, although further refinements to these methods are clearly needed. We conclude that tailoring models to specific cell lines—even with imperfect information—will be a valuable tool for understanding the electrophysiology of iPSC-CMs and the actions of ion channel-blocking drugs.

## Author contributions

Conception—KW, DG, GM, LP; design—KW, DG, GM, LP; data collection—KW, RJ, MH-V, VZ, AA, GS, LP; analysis and interpretation—CL, KW, MC, RJ, DG, GM, LP; writing and review—CL, KW, MC, DG, GM, LP.

### Conflict of interest statement

LP and KW were employed by company F. Hoffmann—La Roche. MH-V, VZ, AA, and GS were employed by Clyde Biosciences. The reviewer DK and handling Editor declared their shared affiliation, and the handling Editor states that the process met the standards of a fair and objective review. The other authors declare that the research was conducted in the absence of any commercial or financial relationships that could be construed as a potential conflict of interest.
