# Supplementary material for: Tailoring Mathematical Models to Stem-Cell Derived Cardiomyocyte Lines Can Improve Predictions of Drug-Induced Changes to Their Electrophysiology
Source: Front Physiol. 2017 Dec 12;8:986. doi: 10.3389/fphys.2017.00986 (PMC5732978; doi:10.3389/fphys.2017.00986)
Supplement: Supplementary file 1 [file DataSheet1.pdf]

Tailoring mathematical models to stem-cell derived cardiomyocyte lines can improve predictions of drug-induced changes to their electrophysiology [Supplementary Materials]

Chon-Lok Lei [1], Ken Wang [2], Michael Clerx [1], Ross H. Johnstone [1], Maria P. Hortigon-Vinagre [4], Victor Zamora [4], Andrew Allan [4], Godfrey L. Smith [4], David J. Gavaghan [1], Gary R. Mirams [3] and Liudmila Polonchuk [2,*]

[1] *Computational Biology, Department of Computer Science, University of Oxford, Oxford, UK*
[2] *Roche Pharma Research and Early Development, Roche Innovation Center Basel, F. Hoffmann-La Roche Ltd., Basel, Switzerland*
[3] *Centre for Mathematical Medicine & Biology, School of Mathematical Sciences, University of Nottingham, Nottingham, UK*
[4] *Clyde Biosciences, BioCity Scotland, Newhouse, UK*

Correspondence*:
Liudmila Polonchuk. Roche Pharma Research and Early Development, Roche Innovation Center Basel, F. Hoffmann-La Roche Ltd., Grenzacher Str.124, 4070 Basel, Switzerland
liudmila.polonchuk@roche.com

1 BIOPHYSICAL MODEL OF IPSC-CM ACTION POTENTIAL

The Paci et al. (2013) model used in this study is based on a classical Hodgkin & Huxley formulation and describes the time-derivative of the membrane potential (V_m) as

$$\begin{aligned} C_m \frac{dV_m}{dt} &= -\sum_j I_j + I_{stim} \\ &= -\,(I_{Na} + I_{CaL} + I_f + I_{K1} + I_{Kr} + I_{Ks} + I_{to} \\ &\quad + I_{NaCa} + I_{NaK} + I_{pCa} + I_{bNa} + I_{bCa} - I_{stim}), \end{aligned} \tag{S1}$$

where C_m is the cell capacitance, I_{stim} is a stimulus current, and the I_j terms describe the various ionic currents. As described in Paci et al. (2013), the model is based on experimental data from Ma et al. (2011), but as no data was available for $I_{NaCa}, I_{NaK}, I_{pCa}, I_{bNa}$ and I_{bCa}, the formulations from a previous hESC-CM model were re-used (Paci et al., 2012) for these currents. Model predictions for 10 major ionic currents are shown in Figure S2.

Each current model I_j has the form

$$I_j = g_{max,j} \cdot g_j(t) \cdot (V_m - E_X), \tag{S2}$$

where $g_{max,j}$ is the maximum conductance, $g_j(t)$ represents the time-dependent opening and closing of the channel and E_X is the appropriate reversal potential. In this study, we left the kinetics terms $g_j(t)$ untouched, and modified only the maximum conductances $g_{max,j}$.

2 SUPPLEMENTARY FIGURES

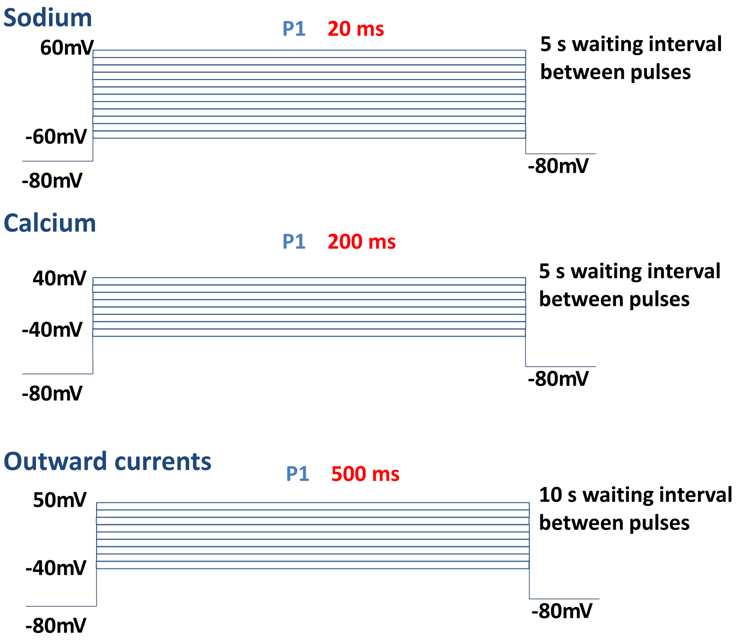

Figure S1. Voltage protocols used for sodium, calcium and lumped outward current measurements.

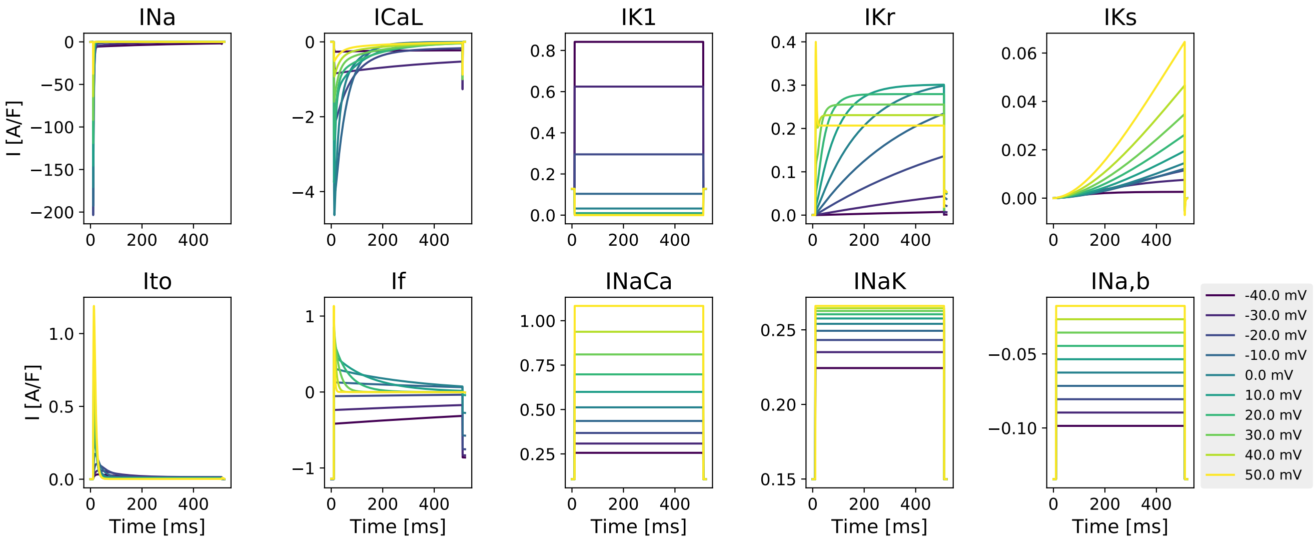

Figure S2. The major ionic currents in the (unaltered) Paci et al. model. All currents were elicited by 500 ms steps from a holding potential of -80 mV to step potentials of -40 mV to 50 mV with 10 mV increments (see Figure S1). Note that, because internal and external concentrations were held constant, I_{NaCa}, I_{NaK} and $I_{Na,b}$ show constant current at a given voltage.

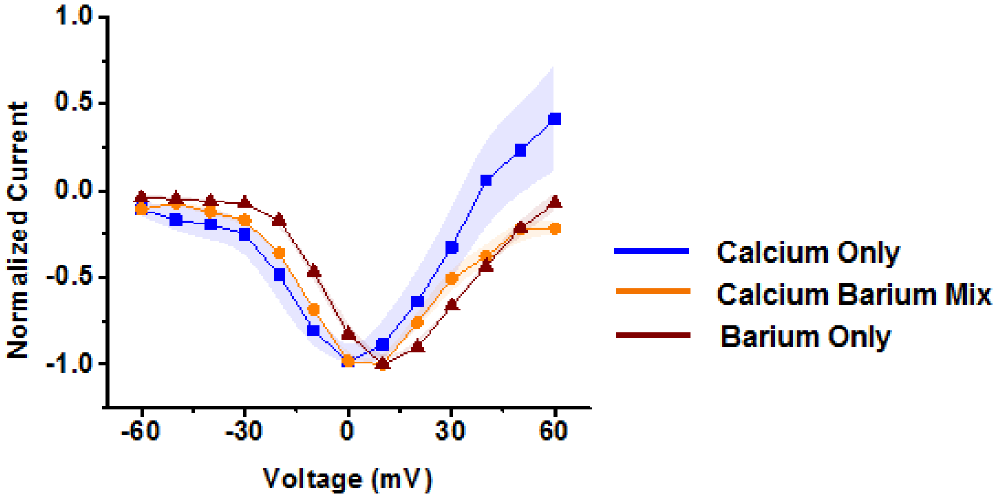

Figure S3. Normalised I_{CaL} current-voltage relationship, measured with Ca^{2+}, Ba^{2+}, and Ca^{2+}/Ba^{2+} solutions. Mean peak currents were −3.1 A/F (Ca^{2+} only, n=8), −2.7 A/F (Ca^{2+}/Ba^{2+}, n=3), and −4.5 A/F (Ba^{2+}, n=3). The experiments used in the main body of our paper were performed using a Ca^{2+}/Ba^{2+} mix. As the figure shows, this can cause a shift in the kinetics towards more positive potentials. However, we used this data only to capture a scaling coefficient, so this shift was not included in our tailored models. Note that, due to between-batch variability in iPSC-CM, it was crucial that we perform all experiments in the same batch of cells, and so calcium-only data from the above figure (recorded later in a different batch of cells) could not be used.

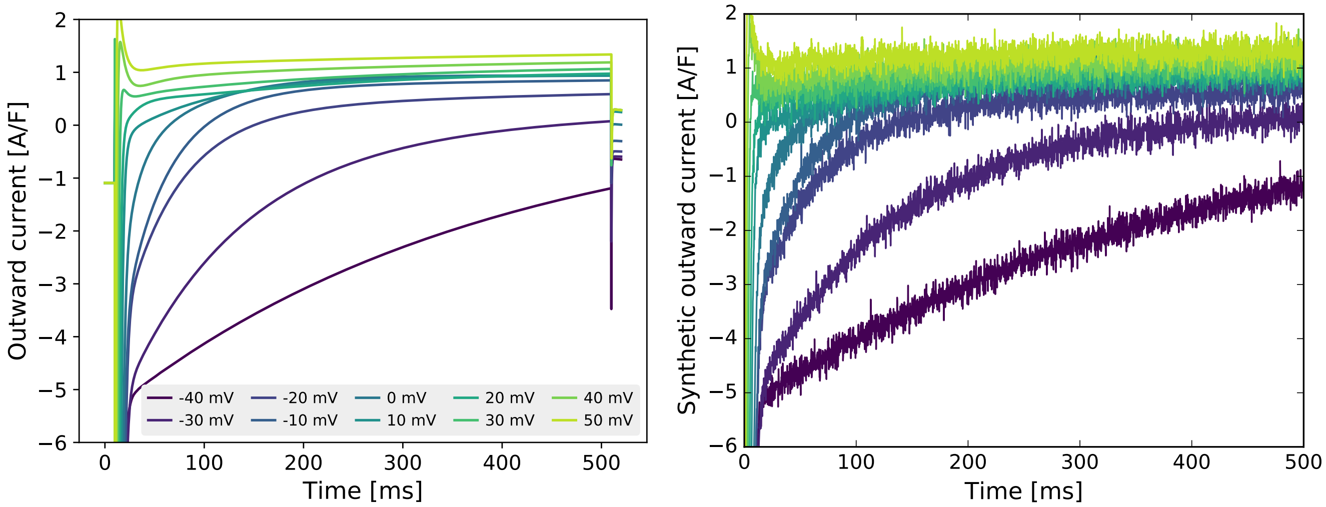

Figure S4. Simulated transmembrane current during the outward protocol (see Figure S1), calculated from the original (untailored) model. **Left:** Without noise. **Right:** With Gaussian noise of $\sigma = 0.15$ A/F.

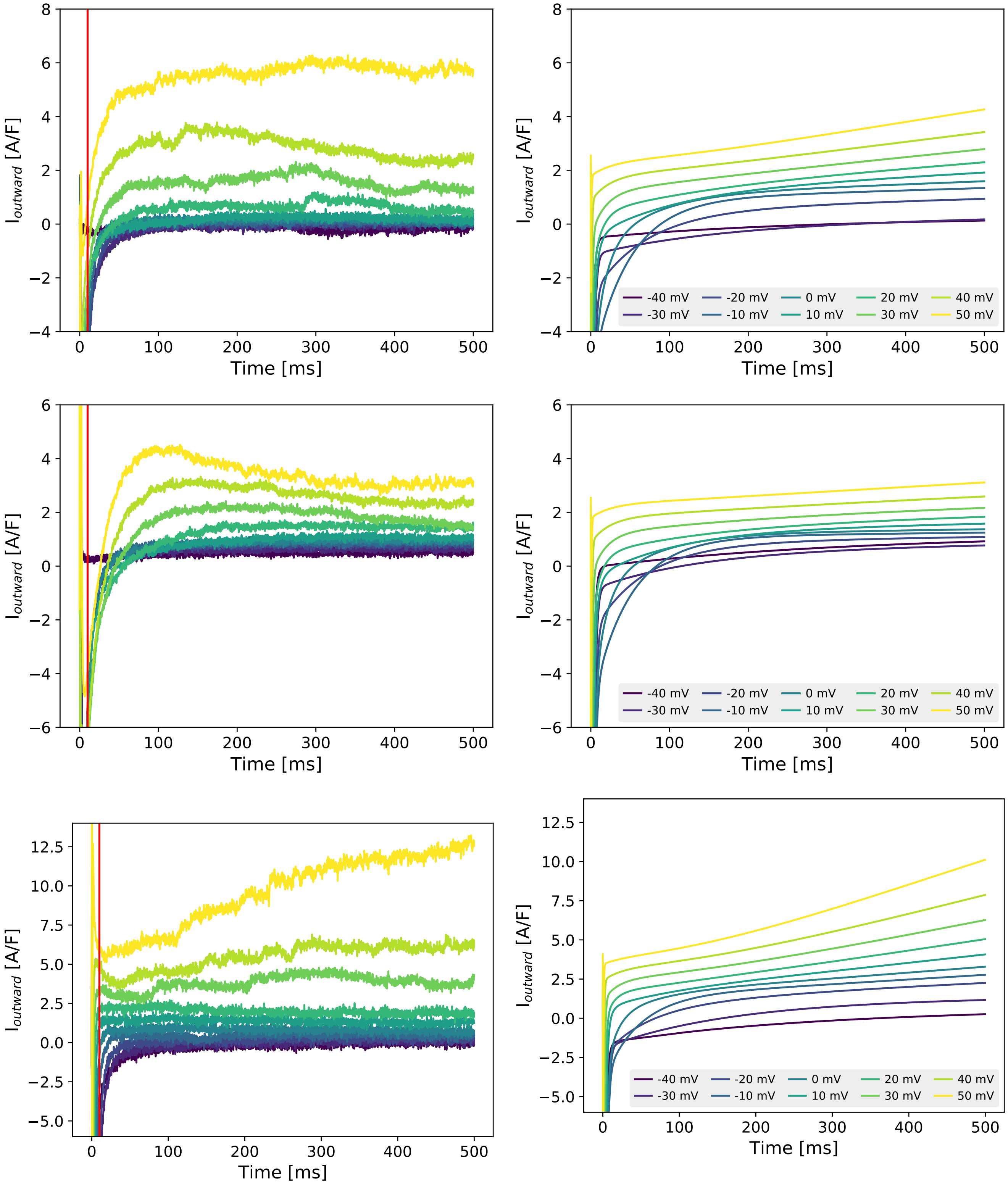

Figure S5. Outward current in three further cells (cells 17, 20, and 21 in Table 2).

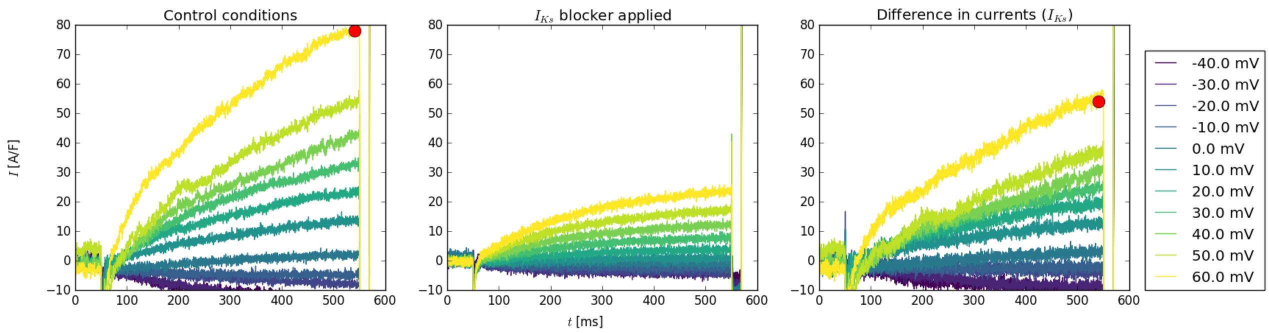

Figure S6. Recording of the transmembrane current of Cor.4U cell using the outward current protocol (see Figure S1). **Left:** control conditions. **Middle:** after drug application of 100 µM Chromanol (an I_{Ks} blocker). **Right:** the difference in the traces which represents the Chromanol-sensitive current (predominantly I_{Ks}). The contribution score c for the measured I_{Ks}, calculated using Equation 1 (i.e. ratio of the indicated red markers), is 69.2, which is indeed comparable to the averaged contribution score of the 22 tailored models 56.3 ± 20.8 (mean $\pm$ standard deviation of the column c under I_{Ks} in Table 2).

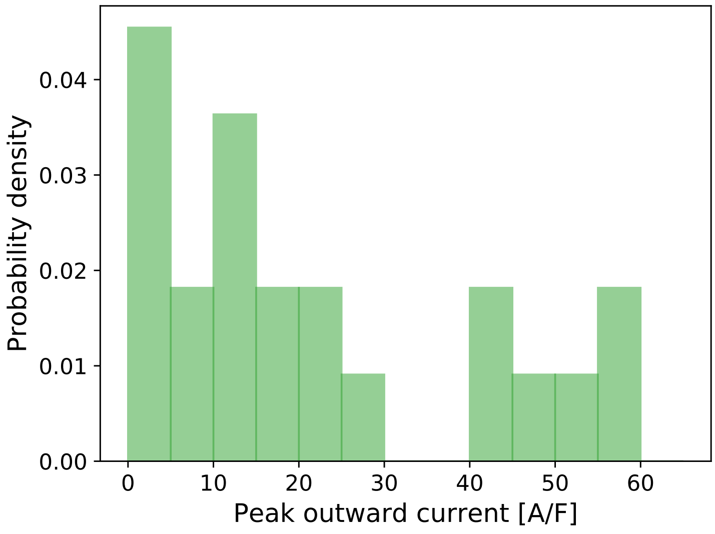

Figure S7. The distribution of peak outward currents in 22 cells.

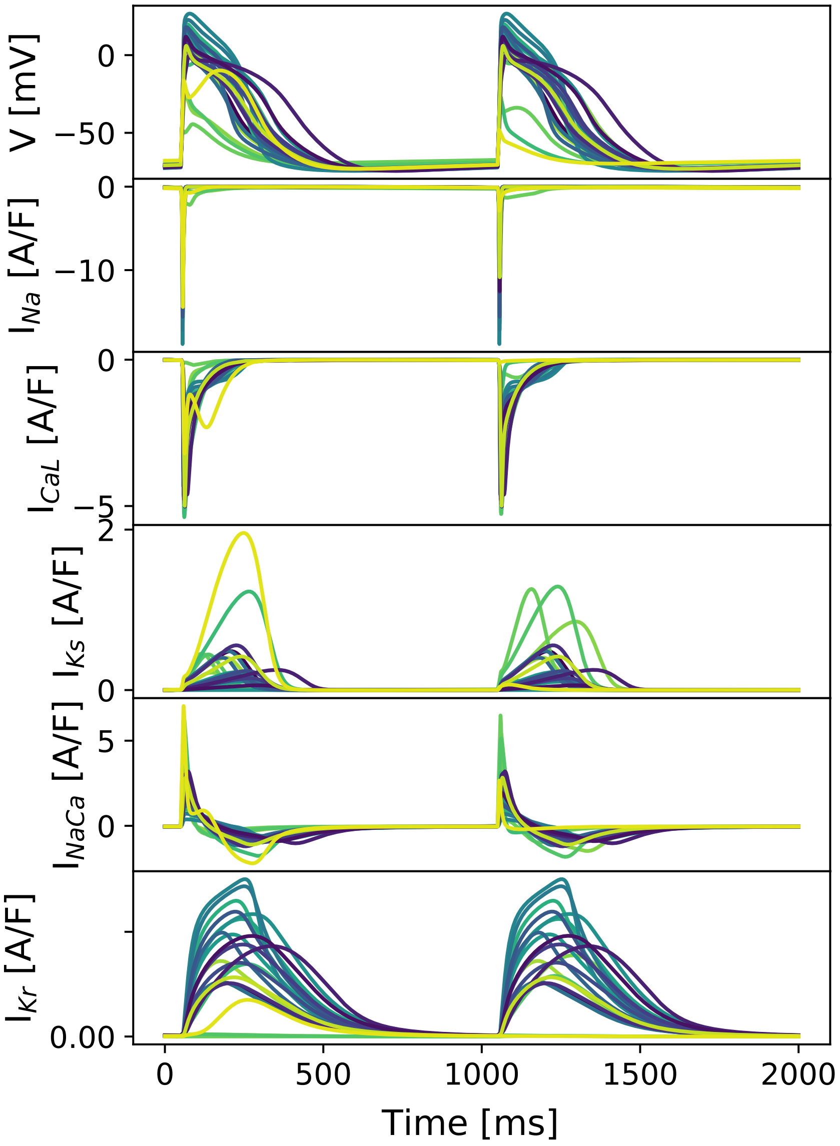

Figure S8. The corresponding contribution of the modified currents I_{Na}, I_{CaL}, I_{Ks} and I_{NaCa}, along with I_{Kr}, throughout the simulated APs for the 22 cells. Note that even I_{Kr} was not modified, because of the feedback of the membrane potential and ionic concentrations, it exhibits variability throughout the 22 cells.

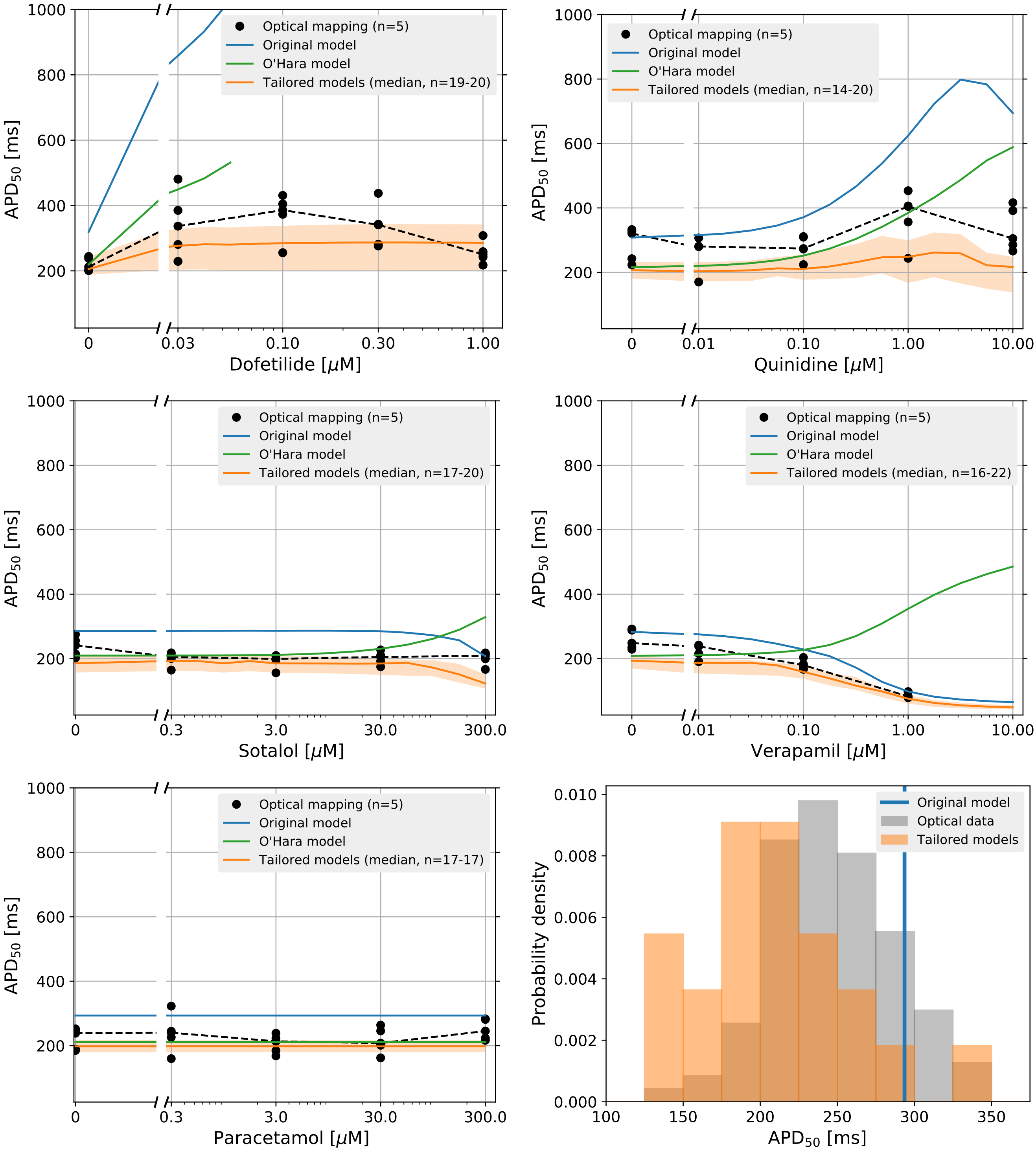

Figure S9. Dose-response curves and histogram of the APD_{50} for reference drugs.

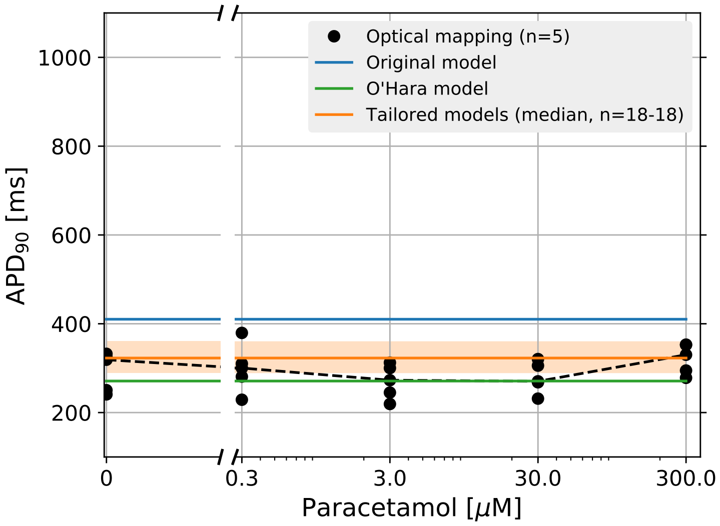

Figure S10. Dose-response curves of the APD_{90} for the control drug paracetamol. All models in the simulations were unchanged for all paracetamol concentration.

3 SUPPLEMENTARY TABLES

	Without noise		With Gaussian noise $\sigma = 0.15$ A/F	
Currents	scaling	contribution	scaling	contribution
I_{Na}	0.99999	0.00000	0.99853	0.00000
I_{CaL}	1.00000	1.29970	1.00247	1.29648
I_{K1}	0.99998	0.00466	1.00642	0.00467
I_{Kr}	1.00004	15.04985	1.01379	15.18145
I_{Ks}	0.99979	4.70086	1.21494	5.68425
I_{to}	0.99986	0.08671	1.05853	0.09135
I_{f}	0.99998	0.00000	1.01297	0.00000
I_{NaCa}	1.00000	78.85821	0.99073	77.74180

Table S1. Synthetic data study. We tested whether our outward current experiment theoretically contains enough information to retrieve the conductances that produced synthetic data from the original Paci et al. model under our outward protocol (see Figure S1), with and without noise (also see Figure S4). The scaling factors are taken with respect to the maximum conductance found in the original Paci et al. model.

	I_{NaCa}		I_{Ks}	
Cell	s	s_{fix}	s	s_{fix}
1	3.32	2.08	139	163
2	2.08	1.19	129	144
3	7.55	6.04	125	156
4	21.1	16.8	467	571
5	6.89	6.15	270	281
6	9.3	7.3	211	255
7	0.951	0.58	37.6	38.9
8	1.54	1.28	19.5	18
9	1.7	0.958	52	63
10	1.67	1.06	—	6.89
11	0.38	0.0124	31.5	32.7
12	0.696	0.338	18.6	19.6
13	3.05	1.93	153	174
14	1.28	—	125	150
15	1.19	0.621	38.3	44.9
16	3.02	1.68	47.3	74.2
17	2.35	1.73	27	35
18	4.03	1.86	138	187
19	5.74	6.44	28.9	16.2
20	2.35	1.93	9.18	13.3
21	3.77	3.12	93.7	102
22	13.9	10.3	288	375

Table S2. The scaling factors (s) in Table 2 compared with the scaling factors (s_{fix}) which we only varied I_{Ks} and I_{NaCa} (and using the scaling factors for I_{Na} and I_{CaL} determined in previously), for I_{Ks} and I_{NaCa}. The scaling factors are taken with respect to the maximum conductance found in the original Paci et al. model. As shown, the scaling factors when varying only I_{Ks} and I_{NaCa} conductance are similar to those found when varying multiple currents.

REFERENCES

Ma, J., Guo, L., Fiene, S. J., Anson, B. D., Thomson, J. A., Kamp, T. J., et al. (2011). High purity human-induced pluripotent stem cell-derived cardiomyocytes: electrophysiological properties of action potentials and ionic currents. *Am J Physiol Heart Circ Physiol* 301, H2006–17. doi:10.1152/ajpheart.00694.2011

Paci, M., Hyttinen, J., Aalto-Setälä, K., and Severi, S. (2013). Computational models of ventricular- and atrial-like human induced pluripotent stem cell derived cardiomyocytes. *Ann Biomed Eng* 41, 2334–48. doi:10.1007/s10439-013-0833-3

Paci, M., Sartiani, L., Del Lungo, M., Jaconi, M., Mugelli, A., Cerbai, E., et al. (2012). Mathematical modelling of the action potential of human embryonic stem cell derived cardiomyocytes. *Biomed Eng Online* 11, 61. doi:10.1186/1475-925X-11-61
